# Singlet-assisted diffusion-NMR (SAD-NMR): extending the scope of diffusion tensor imaging via singlet NMR

**DOI:** 10.3389/fchem.2023.1224336

**Published:** 2023-08-04

**Authors:** Giulia Melchiorre, Francesco Giustiniano, Sundeep Rathore, Giuseppe Pileio

**Affiliations:** School of Chemistry, University of Southampton, Southampton, United Kingdom

**Keywords:** diffusion NMR, diffusion tensor imaging, long-lived spin order, singlet spin order, nuclear magnetic resonance

## Abstract

In this study, long-lived nuclear singlet order methods are combined with diffusion tensor imaging with the purpose of characterizing the full diffusion tensor of molecules diffusing freely in large pores of up to a millimeter in size. Such sizes are out of reach in conventional diffusion tensor imaging because of the limitations imposed by the relaxation decay constant of the longitudinal magnetization. A singlet-assisted diffusion tensor imaging methodology able to circumvent such limitations is discussed, and the new possibilities that it offers are demonstrated through simulation and experiments on plastic phantoms containing cylindrical channels of 1 mm in diameter.

## 1 Introduction

Brownian diffusion is a form of molecular translational motion in which molecules travel through space in a random way as dictated by intermolecular collisions, thermal energy, and the structural boundaries of the space they travel within. Measurements of such motion can be used to make important deductions concerning both the diffusing species itself and its surroundings (Price, 2009; Callaghan, 2011). The most basic value that can be measured to characterize this form of motion is the molecular self-diffusion coefficient, a quantity that measures the extent of molecular translation through Brownian motion in an isotropic space. Molecular diffusion (the prefix “self-” is dropped hereafter, but is implied in all instances in which we refer to diffusion in this paper), however, is not always isotropic; this is the case, for example, when the size and shape of a container impose confinement on molecules that move within it, or when molecules retain some sort of positional order, as in a liquid crystalline phase. To characterize those situations, two quantities, namely, the structural length $l_{S}$ and the diffusion length $l_{D}$ , are commonly introduced. $l_{s}$ reflects the average dimension of confinement (for example, the average diameter of spherical pores or the average length of channels in a structure), whereas $l_{D}$ represents the average distance traveled by the molecules during the diffusion time Δ , also known as the root mean square displacement, $l_{D}=\sqrt{2D\Delta }$ , with *D* representing the diffusion coefficient. These quantities are of particular importance in the study of porous media, which are heterogeneous materials characterized by a matrix hosting a network of voids referred to as pores. The size and shape of such pores can restrict molecular diffusion so that the diffusive motion itself becomes anisotropic, i.e., so that it differs when measured along different directions in space. In these systems (and many others), it is generally more correct to discuss diffusion in terms of a diffusion tensor and to refer to the isotropic diffusion coefficient as the trace of the diffusion tensor. As discussed below, the diffusion tensor can also be diagonalized to reveal the principal directions of diffusion (the tensor eigenvectors) and the diffusion coefficients along those principal directions (the tensor eigenvalues).

Molecular diffusion can be encoded in nuclear magnetic resonance (NMR) experiments (Callaghan, 2011), and NMR measurements of diffusion have already been used to obtain a plethora of molecular and structural information on a variety of systems, spanning from the domain of material sciences (rocks, bones, etc.) to medicine (blood cells, intercellular space, and brain fibers) (Hurlimann et al., 1994; Kuchel et al., 1997; Price et al., 1997; Price et al., 1999; Bullitt et al., 2003; Thorat et al., 2009; Park et al., 2012; Gobel et al., 2017). In particular, and with relevance to this paper, the full diffusion tensor can be measured using the magnetic resonance technique known as diffusion tensor imaging (DTI) (Mori and Tournier, 2013). In DTI, the full diffusion tensor is reconstructed after measurement of molecular diffusion along a minimum of six directions, chosen so as to evenly probe the space around the molecule (Basser et al., 1994; Momot et al., 2011; Mori and Tournier, 2013). There are many spectacular applications of the DTI technique *in vivo* in the medical field, and the most striking of these is perhaps brain tractography (Basser et al., 1994; Pierpaoli et al., 1996; Basser and Jones, 2002).

All magnetic resonance-based diffusion methods (the most famous of which are based on the pulsed-gradient spin echo (PGSE) or pulsed-gradient stimulated spin echo (PGSTE) pulse sequences) have a common approach: a pulsed magnetic field gradient (PFG) imposes a difference in the Larmor frequency of spins along a given direction, thus marking the positions of molecules along that dimension of space; subsequently, molecules (and the spins they carry) undergo Brownian motion for a given time interval, referred to as the diffusion time; finally, a further PFG “reads out” the new molecular positions, revealing the extent of diffusive motion they have undertaken, reflected by a change in signal intensity. It is, therefore, evident that the positional information encoded by the first PFG must survive throughout the diffusion time in order to be decoded by the second PFG. Most typically, NMR-encoded information persists for a maximum time that is of the order of T_1_, the relaxation decay constant of longitudinal spin order. Therefore, T_1_ sets a limit on the diffusion timescale and, hence, on the diffusion space-scale. Important structural information remains hidden, or incorrect conclusions can be drawn, if molecules are not allowed to properly explore the surrounding space. In DTI, for example, diffusion would still appear isotropic (having the same diffusion coefficient in all directions) even when molecules diffused in a highly anisotropic structure made by long and narrow channels if T_1_ only allowed for molecules to move an average distance that is much smaller than the smallest spatial dimension of the space they were filling. With a typical T_1_ value of seconds and a diffusion coefficient of the order of 10^–9^ m^2^ s^−1^, molecules usually travel an average of 10–200 μm during a conventional NMR diffusion experiment. This distance specifies the maximum dimension of the structures that can be accurately studied with conventional NMR diffusion techniques.

In systems consisting of two coupled spin-1/2 nuclei, spin order can be prepared in the form of singlet order, which is a form of nuclear spin order that survives longer than T_1_, most typically by an order of magnitude or more (Pileio, 2020). Molecules containing such a spin system can therefore be used as probes of molecular diffusion in systems where motions occur on a long timescale or over large distances. Indeed, singlet order has already been combined with NMR diffusion experiments to measure very slow flow (Pileio et al., 2015), to image diffusion over a macroscopic scale or probe millimeter-sized confinements with q-space diffraction techniques (Pileio and Ostrowska, 2017), and to measure tortuosity in porous media (Tourell et al., 2018). We group all these methods, in which long-lived spin order is exploited in diffusion experiments, under the acronym SAD-NMR, which stands for singlet-assisted diffusion nuclear magnetic resonance.

In this paper, we discuss a further application of SAD-NMR, with the aim of extending the scope of conventional DTI techniques to allow measurements of the diffusion tensor in porous structures containing pores and channels with sizes on the order of millimeters.

With the intention of discussing the technique and assessing its novel limitations, we present experimental results obtained on 3D-printed model structures containing channels of 1-mm ID.

## 2 Methodology

### 2.1 Basics of DTI

In diffusion tensor imaging studies, molecular diffusion in anisotropic environments is described by a diffusion tensor D , which is a rank-2 tensor of the form:

$$D=\left(\begin{array}{ccc} D_{xx} & D_{xy} & D_{xz}\\ D_{yx} & D_{yy} & D_{yz}\\ D_{zx} & D_{zy} & D_{zz} \end{array}\right).$$ (1)

The diffusion tensor is symmetric ( $D_{ij}=D_{ji}$ ) and therefore contains only six independent values: $D_{xx},D_{xy},D_{xz},D_{yy},D_{yz},D_{zz}$ . These values can be measured, and the full tensor characterized, if molecular diffusion is measured along at least six independent directions in space.

**FIGURE 1:**
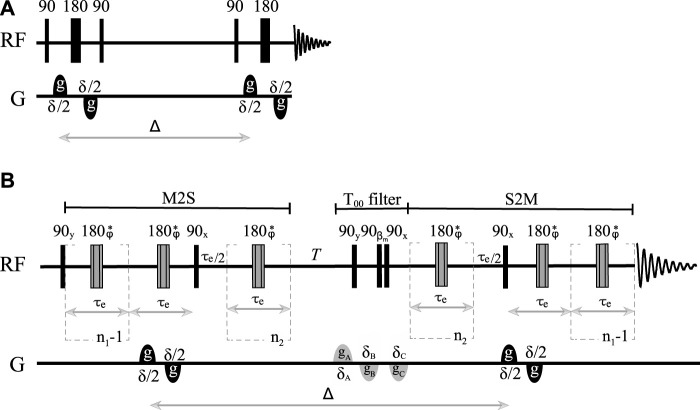
Sketch of **(A)** the pulsed field gradient stimulated echo pulse sequence with bipolar gradients (PFG-STEbp) and **(B)** the singlet-assisted diffusion pulse sequence with bipolar gradients and singlet order filter. $n_{1}=\pi J/\left(2\Delta \nu \right)$ and $n_{2}=n_{1}/2$ . $\tau_{e}=1/\left(2\sqrt{J^{2}+{\Delta \nu }^{2}}\right)$ . $\beta_{m}$ represents the magic angle, and * indicates a composite 180° pulse built as 90_x_180_y_90_x_ and with an overall phase ϕ cycled within each echo train as $\left[x,x,\bar{x},\bar{x},\bar{x},x,x,\bar{x},\bar{x},\bar{x},x,x,x,\bar{x},\bar{x},x\right.$ ].

Diffusion coefficients along any given direction are routinely measured in NMR using one of the many pulse sequences developed over the years, the most basic of these being the pulsed-gradient spin echo cited above. Modifications of this experiment introduced in order to prolong the diffusion time (PGSTE), correct for eddy currents (PGSTEbp), and compensate for thermal convection (PGdSTE and PGdSTEbp) have been available for some years and are widely used by the NMR community (Callaghan, 2011). The results obtained in this work should be compared with those obtained using the PGSTEbp pulse sequence (see Figure 1A), which provides access to the longest diffusion time among all conventional (i.e., non-singlet-based) DTI techniques. As derived by Stejskal and Tanner (1965), PGSTEbp experiments produce an NMR signal whose intensity follows the equation:

$$S=S_{0}\;e^{-\gamma^{2}g_{\alpha }^{2}\delta^{2}D_{\alpha }\left(\Delta -\frac{\delta }{3}-\frac{\tau }{2}\right)}.$$ (2)

The signal is a function of the direction ( α ), total duration ( δ ), and strength ( $g_{\alpha }$ ) of the gradient; the diffusion time ( Δ ); and the diffusion coefficient along that direction ( $D_{\alpha }$ ). $S_{0}$ is the signal when $g_{\alpha }=0$ and τ is the duration of the echo. Most commonly, once a direction is chosen, the diffusion time is fixed and a series of experiments is performed by varying the gradient strength while keeping its duration fixed within the limit δ≪Δ (the condition under which Eq. 2 was derived). Alternatively, it is possible to fix the strength and vary the duration, or to fix both the strength and the duration and vary the diffusion time. In order to derive the diffusion coefficient along the α−th direction, Eq. 2 is fitted to the areas of the acquired NMR peaks.

To reconstruct the whole diffusion tensor, the PGSTEbp experiment (or any alternative) is run along (at least) $n_{d}=6$ independent directions. To interpret the entire dataset when multiple directions are chosen, the equation for the NMR signal is rewritten as follows:

$$S_{g_{\alpha }}=S_{0}\;e^{-\gamma^{2}\delta^{2}g_{\alpha }^{2}\left(\Delta -\frac{\delta }{3}-\frac{\tau }{2}\right)\alpha^{T}D\alpha },$$ (3)

where $\alpha =\left\{\alpha_{x},\alpha_{y},\alpha_{z}\right\}$ now represents a unit vector that specifies the direction of space along which the pulsed field gradient is applied, and the superscript ^*T*^ indicates its transpose.

Note that the choice of the six directions along which diffusion is measured and from which the diffusion tensor is reconstructed is arbitrary so long as they are independent of each other. However, it is best if these directions are chosen to sample the 3D space as uniformly as possible (Jones et al., 1999). The number of directions can also be increased to more than six; this improves accuracy at the expense of experimental time. If the gradient strength is incremented in $n_{g}$ steps per direction, a total of $n_{d}*n_{g}$ areas of experimental signal will be available. The linearized version of Eq. 3 results in a linear system of $n_{d}*n_{g}$ equations that can be matched to the experimental signal areas and solved simultaneously to yield the six unknown components of the diffusion tensor. The diffusion tensor thus reconstructed is expressed in the laboratory frame since the gradient pulses are applied along the laboratory frame directions.

**FIGURE 2:**
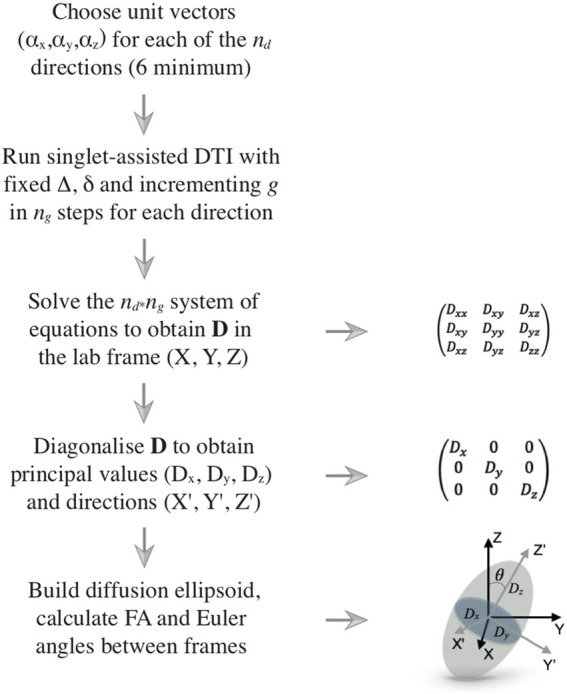
Flow diagram illustrating the DTI procedure. The left column shows the logical flow of operations, starting from the choice of the directions along which the diffusion is measured and ending with the processing of the results, which are typically rendered in terms of a diffusion ellipsoid and its fractional anisotropy. The right column shows several details of the various steps.

The diffusion tensor is diagonalized to obtain its principal values (*D_x_, D_y_, D_z_*) and principal axes ( $X^{\prime },Y^{\prime },Z^{\prime }$ ) as the eigenvalues and eigenvectors of the tensor, respectively. The principal values represent the extent of diffusion along the principal directions. The orientation of the principal frame with respect to the laboratory frame (always expressible in terms of 3 Euler angles) can provide insight into the existence, orientation, and geometry of compartments where diffusion is facilitated in some directions rather than others. This is conveniently captured by drawing the ellipsoid that describes the principal diffusion tensor. The three main axes of this ellipsoid point along the three principal directions (eigenvectors), whilst the elliptic radius along those principal directions is equal (or proportional) to the corresponding eigenvalue. The full procedure is summarized in Figure 2.

If diffusion is identical in all directions of the space, the diagonal diffusion tensor has three identical eigenvalues and its associated ellipsoid is, in fact, a sphere. If diffusion is equal in two directions and faster in the third, the ellipsoid is prolate, with its main axis pointing along the direction of faster diffusion. Similar reasoning can be used for other cases. A crucial point to take into consideration is that the shape of the ellipsoid is connected to the shape of the container inside which diffusion is happening (the pores or channels inside a porous medium, for example). However, for the confinement to become influential and its structural features to be correctly reflected by the diffusion ellipsoid, molecules need to diffuse far enough to feel the restrictions of the container. Recalling the structural and diffusion length previously introduced, the diffusion ellipsoid would appear spherical whenever $l_{D}\ll l_{S}$ . Since for a given molecule diffusing in a structure both D and $l_{S}$ are fixed, the parameter that must be adjusted in order to correctly probe the structure is the diffusion time Δ . The longer the diffusion time is, the bigger the characteristic structural lengths that can be probed with meaningful results. In this paper, we propose an alternative to the PGSTEbp experiment that granted access to much longer diffusion times.

Finally, information about the shape of the diffusion tensor is often conveyed in the form of a single index known as fractional anisotropy (FA), calculated as follows (Basser and Pierpaoli, 1996):

$$FA=\sqrt{1-\frac{D_{x}D_{y}+D_{x}D_{z}+D_{y}D_{z}}{{D_{x}}^{2}+{D_{y}}^{2}+{D_{z}}^{2}}.}$$ (4)

The fractional anisotropy value ranges from 0 to 1, with 0 obtained in the case of isotropic diffusion and 1 in a fully anisotropic structure such as a long, thin channel.

### 2.2 Singlet-assisted DTI

As briefly introduced above, long-lived spin order can provide access to very long diffusion times. This form of order has already been exploited in this respect to measure small diffusion coefficients (Sarkar et al., 2008; Ahuja et al., 2009) or slow flow (Pileio et al., 2015). Here, we aim to combine the latest methodologies for the manipulation of this form of spin order with the more conventional DTI technique discussed earlier. Our proposed methodology is based on the pulse sequence illustrated in Figure 1B (pulse sequence code, in the Bruker TopSpin language, is available upon request) and has been labeled as SAD-TI (singlet-assisted diffusion tensor imaging). In our approach, as in PGSE and PGSTE, the well-studied (Pileio, 2017) singlet preparation (M2S) and reading (S2M) pulse sequence blocks have been sensitized to molecular diffusion with the introduction of a bipolar pulsed field gradient. Specifically, two opposite-in-sign field gradients are placed before and after the 180° radiofrequency pulse within the first echo train of the M2S block. From that time point onwards, molecular position is encoded until a second identical bipolar gradient is applied after the diffusion time Δ , as shown in Figure 1B. At the end of the M2S block, diffusion-sensitized magnetization has been converted to diffusion-sensitized long-lived order, which provides access to much longer diffusion times than those available with spin-echo or stimulated-echo experiments, where diffusion encoding is performed on transverse and longitudinal order, respectively. This is because the decay time constant of singlet order, T_S_, is often more than an order of magnitude longer than the decay time constant of transverse order (T_2_, exploited in PGSE) and longitudinal order (T_1_, exploited in PGSTE). The values of $n_{1}$ , $n_{2}$ , and $\tau_{e}$ occurring in the M2S/S2M blocks are indicated in the caption of Figure 1.

It is worth noting that the minimum Δ accessible with this method is limited by the cumulative duration of the part of the M2S after the bipolar gradient, the duration of the singlet filter, and the initial duration of the S2M until the gradient. This minimum time is of the order of several hundreds of milliseconds. Moreover, the maximum amount of initial polarization that is detectable after the sequence M2S–filter–S2M is theoretically limited to 2/3 (Pileio, 2017) (and in practice generally found to be ∼1/2 because of pulse imperfections and T_2_-driven losses during the echo trains), and this signal loss must be weighed against the benefits of accessing a much longer timescale. In order to obtain the six independent components of the diffusion tensor in the laboratory frame in the SAD-TI experiment, Eq. 3 is fitted to the signal areas recorded in a series of experiments run at different values of gradient strength, one set of gradient strengths for each of the (minimum) six directions, as detailed above.

### 2.3 Errors on diffusion tensors and related quantities

In order to estimate the errors on the principal values and direction of the diffusion tensor, as well as on fractional anisotropy and all other quantities, we used a Monte Carlo approach implemented in a custom-made Mathematica notebook. Note that an analytic approach is also available (Poonawalla and Zhou, 2004) but here not used because unfamiliar to us. Our procedure runs as follows:

1. The standard deviation of each of the six independent diffusion coefficients is extracted from the fitting routine (we use the *NonlinearModelFit* routine in Mathematica).
2. A new diffusion tensor is built by randomly choosing a value for each of its six components using a normal distribution centered at the parameter’s best-fit value; its standard deviation is derived in step 1.
3. The new diffusion tensor is diagonalized, and eigenvalues and eigenvectors are stored in separate arrays.
4. The fractional anisotropy value and the angle between the eigenvector corresponding to the larger eigenvalue and the z-laboratory axis are calculated and stored in separate arrays (any other quantity of interest can be derived in the same way).
5. Steps 1–4 are repeated N times (50,000 in this paper).
6. The average and the standard deviation of the arrays derived via steps 3–5 (containing N sets of eigenvalues, eigenvectors, fractional anisotropies, and Euler angles) are computed and reported.

## 3 Materials and methods

### 3.1 The molecular probe

**FIGURE 3:**
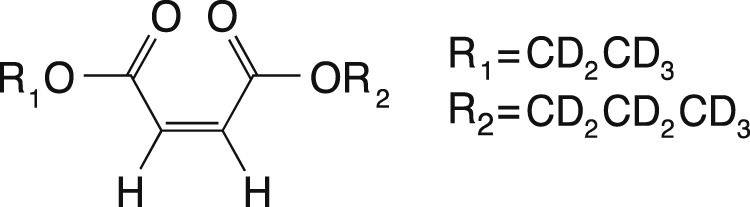
Molecular scheme of 1-(ethyl-d_5_) 4-(propyl-d_7_) (Z)-but-2-enedioate, used in this study as a singlet-bearing molecular probe for singlet-assisted DTI experiments.

The SAD-TI method requires a molecular probe that supports long-lived spin states so that it can travel for long durations and long distances within the large channels and pores of the structure to probe its anisotropy. In our laboratory, we have custom-designed a variety of probes that suit this purpose, and for the current investigations, we used the molecule of 1-(ethyl-d_5_),4-(propyl-d_7_)(*Z*)-but-2-enedioate (EPM) sketched in Figure 3.

The two protons on the double bond constitute the singlet pair, whereas all the other protons have been substituted by deuterons to minimize out-of-pair dipolar relaxation contributions and prolong the singlet lifetime. The difference in chemical shift frequency between the two protons is 3.1 ppb (0.93 Hz in our 7.04 T magnet), and their mutual scalar coupling constant is 11.9 Hz. These values qualify the magnetic properties of the molecular probe as a nearly equivalent spin system. In such systems, singlet order is a good eigenvalue of the high-field spin Hamiltonian and remains long-lived without the need for singlet-locking irradiation, which would not be compatible with diffusion experiments because it can generate heat and related convective flow. The isotropic diffusion coefficient for this molecule, measured in an isotropic liquid sample prepared as a 0.25 M solution of EPM in acetone-d_6_ in a 10-mm OD NMR tube, is $D_{0}=1.6\times 10^{-9}$ m^2^ s^−1^ (obtained using a standard convection-compensated PGdSTEbp pulse sequence).

### 3.2 Structures under investigation

**FIGURE 4:**
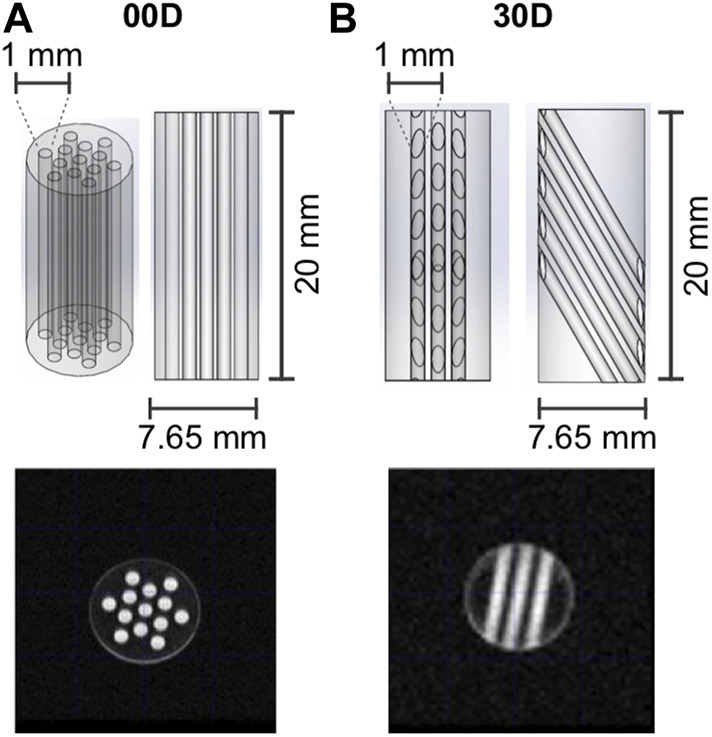
Geometry and dimensions of channel structures **(A)** 00D and **(B)** 30D, which were used in the experiments reported in this paper. The MRI images were generated with a multi-slice multi-echo (MSME) sequence (FOV 2 × 2 cm^2^, 128 × 128 matrix, slice thickness 1 cm, TE = 5.08 ms). Sagittal slices (not shown) were taken to confirm that all channels were fully filled with the EPM solution.

To demonstrate the potential of the SAD-TI methodology, we measured the diffusion tensor of our probe molecule dissolved in a low-viscosity liquid and contained in the long, narrow cylindrical channels cut into the plastic structures shown in Figure 4. Both structures were machined in-house from a rod of polyoxymethylene (POM; this was chosen because it has good resistance to many common organic solvents and is easy to machine). The outer diameter of each structure was 7.65 mm, and the length was 20 mm. Structure 00D (Figure 4A) contained 13 cylindrical channels of 1 mm diameter. The total volume of the 13 channels was 204 mm^3^. The long axis of the channels was oriented along the long axis of the rod, a fact that we indicate as θ = 0°. The angle θ is intended to represent the angle between the long axis of the channels and that of the rod. However, the manufactured structures were additionally aligned with their long axis parallel to the direction of the static magnetic field. Hence, θ also represents the angle between the long axis of the channels and the static magnetic field. Structure 30D (Figure 4B) had 15 cylindrical channels of 1 mm diameter oriented such that their long axis formed an angle θ = 30° with respect to the long axis of the rod. The total volume of the empty channels in structure 30D was 127 mm^3^. Both plastic structures were held within a 10-mm OD (7.8 mm ID) medium-wall LPV NMR tube. The volume of the annular cylinder arising between the tube ID and the plastic OD was 36 mm^3^, and molecules trapped in this region of space experienced a different form of constriction to those trapped in the cylindrical channels.

Structures 00D and 30D were imbibed in 350 μL of a 0.25 M solution of EPM in acetone-d_6_. The NMR tubes were then degassed through 10 cycles of freeze–pump–thaw to minimize the O_2_ content and hence prolong relaxation times. MRI images of the tubes were taken to confirm that all channels were properly filled with the EPM solution (see Figure 4).

### 3.3 Instrumentation

All experiments were run on an Oxford Inst. 7.04 T Magnet coupled to an Avance III Bruker NMR console. This instrument is equipped with a Bruker MIC5 microimaging probe carrying a 10-mm ^1^H/^13^C resonator and a 3-axis gradient system able to deliver pulsed field gradients of up to 1.5 T m^−1^. The samples were maintained at room temperature (21°C), and the probe’s temperature controller was turned off in order to achieve a more uniform sample temperature and minimize convection flow, although thermal convection is here negligible given the relatively small diameter of the channels.

### 3.4 Numerical simulations

In this paper, we present numerical simulations for comparison with experimental data. The routines developed to calculate the diffusion tensor in the structures discussed in Section 3.1 were written in Mathematica (simulation code available upon request). Simulations were based on a simple random-walk approach comprising the following steps (for each structure and each value of diffusion time):

1. The actual sample shape and geometry are reconstructed using the concept of *Region* in Mathematica.
2. The experimentally measured isotropic diffusion coefficient D_0_ and the diffusion time *Δ* used in the experiment are entered.
3. The total number of molecules is set to *N*_*M*_ = 10,000.
4. The total number of random steps is set to *N*_*J*_ = 10,000. This represents the number of steps that each molecule takes during the diffusion time *Δ*; hence, the time step (how long a step lasts) is derived as *t*_*s*_ = Δ/*N*_*J*_.
5. An array of *N*_*M*_ initial molecular positions *r*_*i*_ = (*x*_*i*_ *, y*_*i*_ *, z*_*i*_) is randomly generated such that all molecules lie within the voids of the structure (i.e., within the channels or the annular cylinder formed between the inner wall of the tube and the outer wall of the plastic rod).
6. Starting from *r*_*i*_, the molecular position of each of the *N*_*M*_ molecules is propagated for *N*_*J*_ steps, each step taking a length *l*_*s*_ = (6 D_0_ *t*_*s*_)^1/2^. At each step, the new molecular positions are checked to verify that they fall within the voids of the structure, and if not, a new random step is taken. This process results in an array containing all final positions *r*_*f*_ = (*x*_*f*_ *, y*_*f*_ *, z*_*f*_) for each of the *N*_*M*_ molecules.
7. The αβ component of the diffusion tensor ( $D_{\alpha \beta }$ ) is derived as follows:

$$D_{\alpha \beta }=\frac{1}{2\;\Delta \;N_{M}}\sum_{k=1}^{N_{M}}\left(\alpha_{f,k}-\alpha_{i,k}\right)\left(\beta_{f,k}-\beta_{i,k}\right),$$

with $\alpha ,\beta \in \left[x,y,z\right]$ , *k* being an index that runs on the number of molecules, and the subscripts *i* and *f* indicating the initial and final positions, respectively.

8. Once the six independent ${D_{\alpha \beta }}^{\prime }s$ are calculated, the full diffusion tensor is constructed and diagonalized, and parameters such as the apparent isotropic diffusion coefficient and the fractional anisotropy value are calculated.

## 4 Results and discussion

Before proceeding with the measurement of the diffusion tensor, we measured the decay constant of the longitudinal and singlet orders, T_1_ and T_S_, respectively, for the two structures investigated here. T_1_ was measured with a standard saturation recovery technique, and T_S_ was measured with the M2S/S2M pulse sequence (Pileio et al., 2010; Tayler and Levitt, 2011). The results are summarized in Table 1. The small differences in these parameters across the two structures were within errors and are most likely due to differences in the quality of the degassing procedure.

**TABLE 1:** Longitudinal and singlet order decay constants measured in the sample investigated in this study.

Sample	T_1_ (s)	T_S_ (s)
EPM in acetone-d_6_ in 00D	22 ± 1	230 ± 10
EPM in acetone-d_6_ in 30D	23 ± 1	260 ± 30

The lifetime of singlet order is, therefore, approximately 13 times longer than that of longitudinal order, in this particular case. Lifetime enhancement factors of up to 140-fold have been observed (Stevanato et al., 2015).

To demonstrate the advantages of the SAD-TI procedure and define its limits, we ran the pulse sequence shown in Figure 1B on both 00D and 30D structures. For each structure, experiments were repeated at four different values of diffusion time Δ (namely, 1.5, 30, 120, and 240 s), with the purpose of highlighting the limits of conventional DTI against the benefits of SAD-TI. For each value of Δ , the gradient strength g was incremented in eight steps, linearly spaced within the limits indicated in Table 2. The duration of the diffusion-sensitizing gradients was kept fixed at δ=320 *μ*s. The strengths and durations of the gradients in the T_00_ filter were $g_{A}=-g_{B}=-g_{C}=45$ mT m^-1^ (3% of maximum), $\delta_{A}=1.0$ ms, $\delta_{B}=1.2$ ms, and $\delta_{C}=2.2$ ms.

**TABLE 2:** Minimum and maximum values of gradient strength for the pulse sequence in Figure 1B, expressed as a percentage of the maximum gradient strength available (1.5 T m^−1^). The last column indicates the number of transients acquired and summed upon acquisition.

Δ (S)	$g_{\min}$ (% of max)	$g_{\max}$ (% of max)	Transients
1.5	1	60	2
30	1	14	4
120	1	6.5	8
240	1	4.7	16

The unit vectors for the six directions were chosen from those suggested by Jones et al. (1999) and are summarized in Table 3.

**TABLE 3:** Unit vectors for each of the six directions used in all experiments.

	d_1_	d_2_	d_3_	d_4_	d_5_	d_6_
$\alpha_{x}$	1	0.447	0.447	0.447	0.447	−0.447
$\alpha_{y}$	0	0.895	0.277	−0.724	−0.724	−0.277
$\alpha_{z}$	0	0	0.850	−0.525	0.525	0.850

The values of the parameters in the M2S/S2M blocks in the pulse sequence were experimentally optimized around the theoretical values and found to be $n_{1}=20$ , $n_{2}=10$ , and $\tau_{e}=41.8$ ms.

The results of the SAD-TI procedure are reported in Table 4. Instrumental differences in the gradient performances along different directions were corrected by independently calibrating the gradients along each direction, such that the SAD-TI experiment with Δ=1.5 s resulted in an exactly spherical (i.e., isotropic) tensor. This was effectively achieved by multiplying each of the unit vectors in Table 3 by a correction factor calculated as follows:

$$c_{i}=\frac{D_{i}}{\overset{n_{d}}{\underset{i=1}{\Sigma }}D_{i}},$$ (5)

where $c_{i}$ is the correction factor for the i^th^-direction, $D_{i}$ is the diffusion coefficient measured along the i^th^-direction in our SAD-TI experiment with Δ=1.5 s, and the term in the denominator is effectively the average diffusion coefficient along the $n_{d}$ directions (six in our case). The success of this calibration procedure results in a perfectly null FA value for the Δ=1.5 s case.

**TABLE 4:** Experimental and simulated results of the SAD-TI procedure on 00D and 30D at different values of diffusion time. The diffusion length $l_{D}$ is calculated using the measured isotropic diffusion coefficient and the actual value of *Δ*. Differences are calculated as experimental minus simulated values divided by experimental values.

		Experiments			Simulations			Difference		
Δ (s)	$l_{D}$ (μm)	$D_{0}^{a}$ (10^–9^ m s^−2^)	FA	θ (°)	$D_{0}^{a}$ (10^–9^ m s^−2^)	FA	*θ* (°)	$D_{0}^{a}$	*FA*	*θ*
00D										
1.5	70	1.66 ± 0.04	0*	65 ± 28	1.45	0.08	2.5	13%	—	—
30	310	1.25 ± 0.05	0.27 ± 0.04	0.9 ± 3	1.17	0.27	0.8	6%	0%	11%
120	620	0.92 ± 0.02	0.59 ± 0.01	0.3 ± 0.1	0.88	0.59	0.3	4%	0%	0%
240	875	0.83 ± 0.02	0.86 ± 0.02	1 ± 0.1	0.72	0.74	0.5	13%	14%	50%
30D										
1.5	70	1.65 ± 0.03	0*	64 ± 26	1.45	0.07	13.4	12%	—	—
30	310	1.27 ± 0.03	0.27 ± 0.02	29.3 ± 2	1.17	0.27	24.7	8%	0%	16%
120	620	0.87 ± 0.06	0.62 ± 0.02	29.3 ± 1	0.85	0.57	27.1	2%	8%	8%
240	875	0.72 ± 0.02	0.84 ± 0.02	32.0 ± 0.4	0.72	0.69	27.0	0%	18%	16%

**FIGURE 5:**
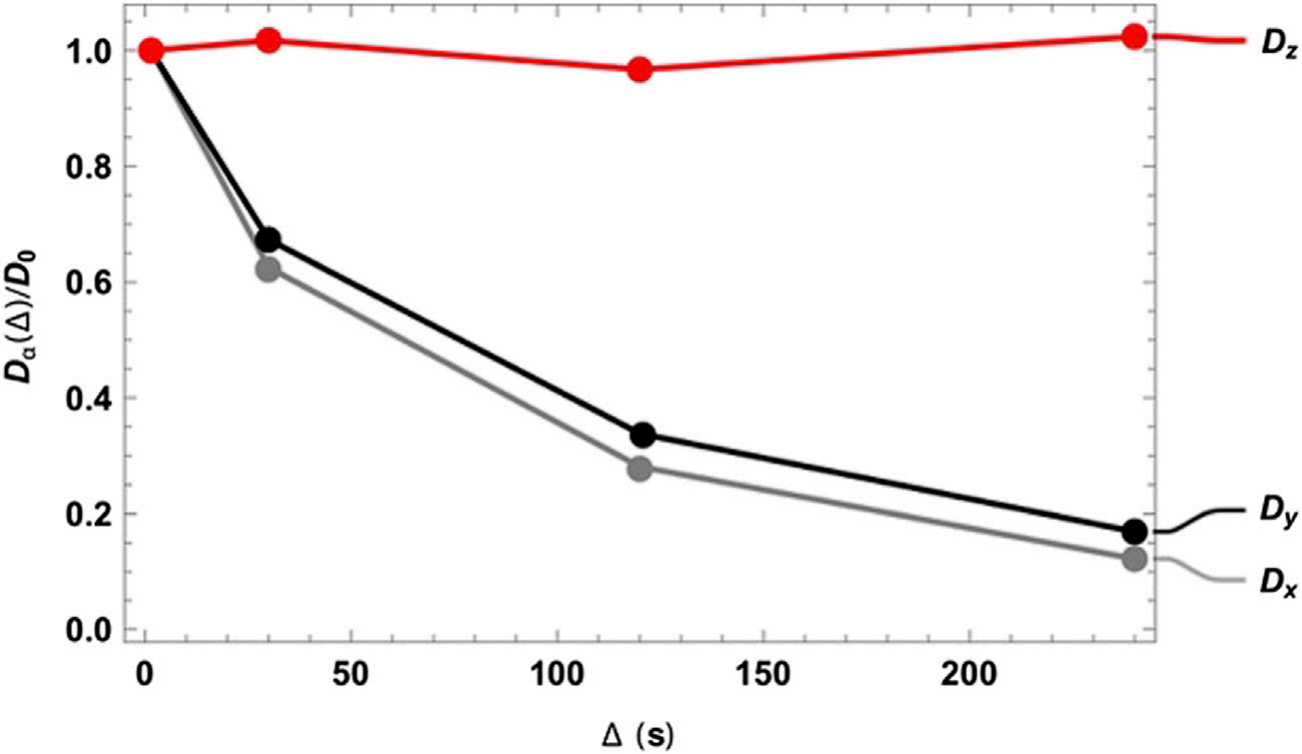
Diffusion constant measured for structure 00D along the three principal directions normalized to the isotropic diffusion constant and plotted against the diffusion time ∆ . The rapid drop observed in the *x* and *y* principal directions reflects restricted motion in that plane. No restriction is observed in the *z* principal direction, since this coincides with the long axis of the channel.

As expected, at very short diffusion times (of the order of a few seconds), it was not possible to measure either the correct fractional anisotropy or the orientation of the channels with respect to the magnetic field. At modestly long diffusion times (of the order of ∼3T_1_), the channel orientation was correctly obtained, although the measured fractional anisotropy was quite far from the expected value of ∼0.95 for a cylinder of 1 mm radius and 20 mm length. Our numerical simulations of the diffusion tensor in the actual geometries were in good agreement with the experimental values, despite the low level of sophistication of the model used to simulate the random walks and the relatively low number of random steps and molecules employed. To obtain a correct value for FA, a diffusion time of several minutes was required. This can be explained as follows: in order to track the channel orientation, it is sufficient merely to identify the direction associated with the largest eigenvalue of the diffusion tensor, no matter the exact value of the diffusion coefficients along each principal direction. For this reason, the channel orientation was correctly measured even at relatively short diffusion times (note that this still required diffusion times of tens of seconds). To properly characterize the FA, the limiting value of the diffusion coefficient along each principal direction must be correctly measured, and for this to happen, a diffusion time that allows for a diffusion length of the order of the characteristic length of the structure must be used. To further highlight the importance of the technique, we plotted the diffusion constant measured for structure 00D along each principal direction at different values of diffusion time, normalized to the isotropic diffusion constant (Figure 5). The plot shows that diffusion is essentially free along the *z*-direction (coinciding with the long axis of the channels) and restricted, to an equivalent extent, along the two perpendicular directions. In a structure with connected pores, the value of $D_{\alpha \beta }\left(\Delta \right)/D_{0}$ tends to be 1/ α for sufficiently long diffusion time Δ , with α representing the tortuosity (Latour et al., 1993), i.e., the ratio of the effective path length to the shortest path length in a porous medium. Tortuosity is, therefore, an indicator of pore connectivity and, as such, a fundamental quantity in understanding fluid transport through the material.

Although tortuosity is not explicitly addressed in this paper, Figure 5 demonstrates how the singlet-assisted DTI technique presented here can provide easy access to the value of tortuosity along the three principal directions of a porous medium with large pores.

To make a fair comparison with other techniques, this same information can be accessed through conventional DTI (PGSTE-based) if a molecule with very long T_1_ or a larger diffusion constant is available. Assuming that relaxation is dominated by a dipole–dipole interaction, as is often the case for small molecules in non-viscous liquids, minutes-long T_1_ values are rare and usually linked to nuclei with a low gyromagnetic ratio, whose NMR sensitivity is often the limiting factor in diffusion studies within porous media. The use of gases, whose self-diffusion coefficients are 4–5 orders of magnitude greater than those of liquids, is a good alternative, since the molecules can move much further, even within a short diffusion time. The use of hyperpolarized-Xe has indeed been proposed for diffusion studies of a similar nature (Mair et al., 2001), but again, the low sensitivity requires hyperpolarization, which adds a level of complication and requires specific expertise and relatively costly equipment. Our technique can easily be implemented using conventional high-resolution NMR hardware, and this constitutes a clear advantage over other techniques.

## 5 Conclusion

We have presented a singlet-assisted version of the well-known diffusion tensor imaging technique that provides access to measurements of the full diffusion tensor of molecules diffusing within porous media with large pores. The diffusion tensor can be used to access structural information such as fractional anisotropy, pore geometry, and orientation, as well as tortuosity, an important parameter that has so far been inaccessible to NMR, since conventional DTI fails to accurately measure it in structures with pores above 50–100 microns in size. Structures containing such large pores include battery electrodes, scaffoldings for tissue engineering, and certain rocks.

## Data Availability

The raw data supporting the conclusions of this article will be made available by the authors, without undue reservation.
